# Blood biomarkers of the late phase asthmatic response using RNA-Seq

**DOI:** 10.1186/1710-1492-10-S2-A61

**Published:** 2014-12-18

**Authors:** Amrit Singh, Casey P Shannon, Gail M Gauvreau, Paul M O'Byrne, J Mark FitzGerald, Louis-Philippe Boulet, Scott J Tebbutt

**Affiliations:** 1 James Hogg Research Centre, St. Paul’s Hospital, University of British Columbia, Vancouver, British Columbia, V6Z 1Y6, Canada; 2 Prevention of Organ Failure (PROOF) Centre of Excellence, Vancouver, British Columbia, V6Z 2K5, Canada Department of Medicine, McMaster University, Hamilton, Ontario, L8S 4L8, Canada; 3 Department of Medicine, McMaster University, Hamilton, Ontario, L8S 4L8, Canada; 4 Vancouver Coastal Health Research Institute, Vancouver General Hospital, Vancouver, British Columbia, V5Z 1M9, Canada; 5 Centre de Pneumologie de L’Hopital, Université Laval, Sainte-Foy, Quebec, G1V 0B4, Canada

## Background

Asthmatic individuals respond differently, but reproducibly, to allergen inhalation challenge. Some individuals develop an isolated early response (ER), while others develop a dual response (DR). Peripheral blood cell transcriptome signatures can discriminate isolated early responders from dual asthmatic responders undergoing allergen inhalation challenge.

## Methods

35 individuals (17 ERs and 18 DRs) participated in the allergen inhalation challenge. Blood samples were obtained prior to and 2 hours post allergen inhalation challenge. HiSeq-Illumina paired-ends 100bp sequencing was performed. A UCSC transcriptome using both the UCSC gene and gene-isoform transcripts was created using the RSEM (RNA-Seq by Expectation Maximization) package. RSEM uses Bowtie to align read files to the reference transcripts and estimates the expected number of counts per transcript. The biomarker pipeline consisted of 20x5-fold deep cross-validation using limma voom (linear models for microarrays and RNA-Seq using variance modeling at the observational level) for differential expression and elastic net for classification. Gene set enrichment analysis was performed using GeneGo.

## Results

Classification performance of the pre-challenge classifier across the 100 panels included an AUC of 0.76±0.02, a sensitivity of 0.70±0.03 and a specificity of 0.72±0.02. There were 511 gene transcripts identified across the 100 panels. Pathway analysis of the 511 gene transcripts identified lectin induced complement pathway, integrin inside-out signaling, alternative complement pathway and function of MEF2 in T lymphocytes as the top ranked pathways. Classification performance of the post-challenge classifier across the 100 panels included an AUC of 0.62±0.002, a sensitivity of 0.67±0.002 and a specificity of 0.54±0.003. There were 301 gene transcripts identified across the 100 panels. Pathway analysis of the 301 gene transcripts identified clathrin coated vesicles formation, CDC42 in cellular processes and regulation of actin cytoskeleton by Rho GTPases as the top ranked pathways.

## Conclusion

The pre-challenge classifier out-performed the post-challenge classifier in the internal deep cross-validation as depicted by the classification performance measures. The lower performance of the post-challenge classifier in discriminating ERs from DRs may be due to a dilution of signal, given that both responder groups are undergoing an immune response to allergen inhalation. Incorporating changes in cellular composition and gene isoform expression estimates may improve classification performance and also reveal new insights into the mechanisms of the late phase asthmatic response.

